# Mind-Wandering Changes in Dysphoria

**DOI:** 10.3389/fpsyt.2020.544999

**Published:** 2020-09-11

**Authors:** Alice Guesdon, François-Xavier Lejeune, Jean-Yves Rotgé, Nathalie George, Philippe Fossati

**Affiliations:** ^1^ Paris Brain Institute, (ICM), UM75, CNRS UMR 7225, Inserm U1127, Sorbonne Université, Paris, France; ^2^ Department of Adults Psychiatry, Pitié-Salpêtrière Hospital, APHP Sorbonne Université, Paris, France

**Keywords:** cognition, mind-wandering, dysphoria, mood, depression

## Abstract

Changes in mind-wandering (MW) and involuntary autobiographical memory (IAM) in dysphoria have been explored with conflicting results. The aim of this study was to evaluate both MW and IAM in a group of 23 stable dysphoric participants compared to 37 controls and to compare their thoughts characteristics (i.e., specificity, visual perspective, time orientation, and emotional valence). To make this study comparable with previous research, we used two different monotonous vigilance tasks (with and without verbal interference stimuli). Our results showed a significantly greater focus on MW thoughts in dysphoria. The characteristics of spontaneous thoughts content did not differ significantly between our dysphoric participants and controls, which is not in favor of strong emotional dysfunction. Our results suggest a difficulty to regulate the occurrence of self-generated thoughts rather than their content, that may confer to dysphoric subjects increased cognitive risk to develop a major depressive episode.

## Introduction

Dysphoria is defined by the presence of depressive symptoms that do not fulfill the diagnostic criteria of a major depressive episode (MDE) but may cause personal suffering in daily life and increased risk for a MDE. Like MDE patients, individuals with dysphoria showed cognitive impairment, especially regarding attention, executive functions, and memory (1–4).

Depressive symptoms may lead to difficulties in retrieving specific information with vivid and emotional details in the autobiographical memory store (5). Dysphoria and MDE are also characterized by increased and faster retrieval of negative emotional memories. However, a literature review provides conflicting results. For instance, Lemogne et al. (6) showed that the reduction of episodic details affects both positive and negative events in acute depressed patients, depending on the presence or absence of intrusive negative memories. On the opposite, depending on the mode of remembering, subjects with depressive symptoms may retrieve information with normal specific and emotional details (7), especially when memory is involuntary recalled. Involuntary autobiographical memory (IAM) refers here to memories coming spontaneously to mind in opposition to memories retrieved with deliberate effort (8). IAM can be assessed with both diary and laboratory methods. Watson et al. (7) showed, using diary assessment, that acute major depression did not change perceived vividness of events during the recall of involuntary autobiographical memories, unlike during the voluntary recall of autobiographical memory events.

Likewise IAM retrieval is usually associated with recall of greater emotional detail calling for more emotional regulation (9) in healthy controls and subjects with high depressive symptoms. This emphasizes the key role of autobiographical memory in psychopathologies associated with impaired emotional regulation such as Post-traumatic Stress Disorder and major depression.

Here, we are interested in the relationships between Mind Wandering and IAM in dysphoria. IAM retrieval is a common phenomenon when our mind wanders. Mind-wandering (MW) refers to the spontaneous flow of thoughts that is detached from our immediate action. Very common, MW takes up to half our waking time (10). MW thoughts can be self-generated or triggered by an external stimulus (as the scent of a madeleine drives Marcel Proust to remember vivid memories) (11) and can have different time-orientations (10). Considering IAM as past oriented MW thoughts, both involve *a priori* the same brain structures, coupling the default mode network (DMN) which includes medial parts of the brain (e.g., prefrontal medial cortex, posterior cingulate cortex, and precuneus) with the fronto-parietal control network (FPCN) (12).

When oriented toward past or future, MW thoughts are a spontaneous kind of mental time travel. Time orientation of thoughts is associated with emotional bias: a more positive emotional bias for thoughts directed toward future than toward past (13, 14). On the other hand, MW competes with the cognitive processes involved with the present action, resulting in detrimental effects on externally oriented cognitive processes (15).

Changes in MW and IAM in subjects with dysphoria have been explored with conflicting results (16–20). Several studies specifically concerned with MW emphasize its increase when depressive symptoms are present (16–18). Others showed that induction of a negative mood in healthy subjects increased the tendency for MW to be past-oriented rather than present or future oriented (16, 18). One would then expect to find more past-oriented MW (more IAM)—and with different phenomenological characteristics (i.e., with more negative content)—in dysphoric subjects compared to controls. However, studies assessing specifically IAM with both laboratory and diary methods (19, 20) found no difference in the frequency and characteristics of IAM between dysphoric subjects and controls.

The first aim of the present study was to evaluate both MW and IAM in the same group of stable dysphoric subjects compared to controls. The second aim was to compare MW focus and characteristics of spontaneous thoughts—time orientation, specificity, perspective taking (field vs. observer) and emotional valence (positive vs. negative)—between subjects with dysphoria and controls. To make this study comparable to different previous studies, we decided to use two different vigilance tasks, one designed to elicit IAM with additional verbal stimuli [the Verbal Interference Monotonous Task (VIMT) (19, 20)] and one similar to tasks usually exploring MW independently of time orientation, without verbal stimuli [Low Demand Task (LDT)] (10, 15, 21).

We hypothesized that: 1) the VIMT would induce more retrieval of IAM compared to the LDT in both dysphoric and non-dysphoric subjects; 2) dysphoric subjects would show increased MW with more past-oriented thoughts (that is, IAM) than control subjects; 3) dysphoric subjects would show thoughts with more negative content and with less specificity compared to non-dysphoric subjects.

## Methods

### Participants

Healthy volunteers were recruited after their application on a website dedicated to scientific research studies (www.expesciences.risc.cnrs.fr) and paid 30 euros compensation for their participation. They were 18 to 50 years old, with neither major neurological or psychiatric history nor psychotropic drug therapy.

In order to define dysphoric and non-dysphoric group, we used an auto-evaluation by the Beck Depression Inventory (BDI). Participants were assigned to the dysphoric group (D) when their BDI score was >16 and to the non-dysphoric group (ND) when it was <7. In order to ensure stability of the depressive symptoms, participant rated the BDI scale both on the website application and the day of the experiment (maximum 10 days after they applied). If the BDI score did not match for the same group on the website application and the day of the experiment subjects were not included in the group analysis.

### Monotonous Attentional Tasks

The set of tasks was coded on MATLAB 2016b with the Psychotoolbox module.

The experiment took place in the morning, at 9 or 11 am, to limit the eventual MW’s variability linked to the moment of the day.

Exploring IAM and MW, consisted of two monotonous computerized tasks coming one after the other without interruption (LDT and VIMT) (**Supplement Material 1**).

Both are simple vigilance tasks with un-frequent targets (on average 2.4 targets per minute) where participant must press the space bar with their right index. Participants were sitting with their eyes at 80 cm from the center of the screen; they were asked if the characters were visible for them.

The LDT is a classical task for exploring MW (10, 15, 21, 22). Numbers from 1 to 10 succeed each other randomly, centered on a black screen in white Arial characters 16. They are displayed 1000 milliseconds (ms) with a dark screen lasting 1000 ms between each number; 3 is the un-frequent target number. In the version used, subjects have to press the space bar only when the number 3 (un-frequent target) appears on the screen.

The VIMT, is based on the task designed by L. Kvavilashvili’s team (University of Hertfordshire, UK) and then adopted in several studies exploring IAM in laboratory and more generally spontaneous mental time travel (19, 20, 23). Slides with horizontal lines succeed each other, the un-frequent target slides are with vertical lines. At the center of each slide of the VIMT, a verbal expression with a negative or neutral positive valence, supposed to stimulate MW toward the past. The written instructions mention to the subjects that they have been designated for the group of subjects who does not have to pay attention to the verbal expressions. As in the LDT, subject has to press the space bar again only when the vertical lines appear on the screen.

### Thought Probes

Each task lasted 15 min in which 9 computerized thought probes are displayed pseudo-randomly.

The thought-probes explore the nature and characteristics of the subject’s thought provided in **Supplement Material 2**.

The thoughts can be linked to the task [on task (OT)], directed to something else [tune out (TO)] or to “nothing” when the subject does not have access to its content [zone out (ZO)] (17).

If thought is directed to something else, the probe asks if the subject was distracted by some perception in the room (noise, heat, etc.) and if the subject was aware of being off task. It also asks if the thought was spontaneous or deliberate.

Time orientation of thoughts is explored by the probe when thought is described as spontaneous and not driven by perceptual distraction. It can be defined as directed toward past, present, future or not time-oriented.

When the thought is directed to something else than the task (TO) and toward the past (IAM) or the future, the probe explores its content characteristics. The participants were asked about the specificity of the thought (if it refers to a single episode, of short duration, well localized in time and space, with details), its perspective (if the subject sees the thought in a field or an observer perspective) and its emotional valence (rather negative or positive). For degree of detail, perspective and emotional valence the participant places a cursor on a line between the two ends of each dimension (very vague/very accurate, field/observer perspective, very unpleasant/very pleasant thought). These dimensional cursor-placed answers are reported as a number between 0.0 and 9.9 for statistical analyses.

### Procedure

Participants were tested 2 to 10 days after the on-line BDI test on the PRISME core facility at the Brain and Spinal cord (ICM) institute. Subjects performed continuously both monotonous attentional tasks in a random order. Subjects completed three questionnaires after the experiment: the BDI scale to check that their score corresponded to the group in which they were included by internet (<7 or ≥ 16), the Ruminative Response Scale (RRS) to evaluate their ruminative profile and the Day Dreaming Frequency Scale (DDFS).

This protocol was validated by an ethics committee and carried out in accordance with the code of Ethics of the World Medical Association (Declaration of Helsinki). The informed consent of participants was obtained prior to the start of the experiment.

### Statistics

Statistical analyses were conducted using the R software (version 3.5.2) (24).

The ordering of the two tasks (LDT and VIMT) was randomly assigned for each participant, regardless of the BDI scores.

#### Comparison Between LDT and VIMT Tasks

We first compared the nature of thoughts during the VIMT and the LDT for all subjects according to the order of task presentation and independently of the BDI score.

Since the experiment provides repeated values for the same individual, the number of thoughts was analyzed with linear mixed effect models (LMMs) including the fixed effects of “task” (LDT and VIMT) as a within-subject factor and “order” as a between-subject factor, and the subject identifier as a random effect. For each type of thoughts (OT, ZO, and TO), a LMM was fitted to the data using the lmer function of the R package lme4. To improve the assumptions of normality and constant variance of residuals, count data were square root transformed prior to analysis. The significant effect of “task” and “order” was then assessed based on type II Wald chi-square tests using the ANOVA function in the R package car.

The Wilcoxon-signed-rank test for paired observations was then used to investigate the difference in proportion of the TO past-oriented thoughts between the two tasks (number of TO past oriented thoughts divided by the total number of spontaneous, non-driven by perception TO thoughts)

#### Comparisons Between the Dysphoric and Non-Dysphoric Groups

Subjects with variation of BDI scores between the internet BDI score and the day of the experiment BDI that induced changes in group allocation were excluded of these analyses, in order to have groups presenting a stable difference concerning depressive symptoms. Likewise subjects with BDI ≥7 and <16 were excluded from this analysis. Demographic data were summarized as mean ± standard deviation (SD) for age and sex, and frequency counts for sex. Significant differences between the two groups were assessed using t-tests (age and education) and a chi-square test (sex).

We evaluated the attentional fluctuations of the LDT and VIMT tasks from the number of errors and the variability of the reaction times. For the behavioral data, we used t-tests between dysphoric and non-dysphoric groups for errors and response time. A Levene test was then applied to compare the variance of RT between the groups. In order to compare the type of thoughts, we compared the two groups with t-tests for the different kind of thoughts (on task/zone-out/tune-out thoughts).

The ratio of past oriented spontaneous thoughts among all spontaneous and non-driven by perception TO thoughts was compared between groups by a Wilcoxon-Mann-Whitney test. The same test was used to compare the ratio of mental-time travel (past and future oriented thoughts combined) between groups.

Finally past and future oriented spontaneous TO thoughts characteristics were compared between dysphoric and non-dysphoric through another LMM.

All linear mixed models were fitted using the lmer function of the R package lme4. Significance of main effects and interactions was then evaluated by Type II Wald chi-square tests using the ANOVA function in the R package car.

The level of statistical significance was set at p < 0.05 for all tests.

## Results

A total of 83 participants were included in the study (mean age = 24.8 years ± 5.4) with 57 women (mean age = 24.2 years ± 4.9) and 26 men (mean age = 26.2 years ± 6.3). Out of these, as initially planned, 21 participants were excluded of the statistical group analysis because the BDI score the day of the experiment was not anymore into the limits of the initial inclusion group. The dysphoric group was composed of 23 participants (5 men and 18 women, mean age = 22.3 years ± 2.3) and 37 participants (13 males and 24 females, mean age = 26 years ± 5.9) were included in the non-dysphoric (control) group.

### Comparison of VIMT and LDT Tasks

We first compared the nature of thoughts during the VIMT and the LDT for all 83 subjects (**Table 1**).

**Table 1:** Tasks comparison (type II Wald Chi2).

Thought probe	LDT	VIMT	II/Wald/χ^2^	p	*	SD-LDT	SD-VIMT
On-task	3.48	3.44	0.0644	0.8	ns	2.44	2.08
Off-task/Tune out	4.14	3.87	1.2107	0.27	ns	2.4	2.41
Off-task/Zone out	1.35	1.69	2.835	0.092	ns	1.81	2.1

*p < 0.05. ns = not significant.

There was no effect of task: no significant difference of each kind of thought between LDT and VIMT (OT: χ^2^ = 0.0644, p = 0.800; TO: χ^2^ = 1.2107, p = 0.271; ZO: χ^2^ = 2.835, p = 0.092).

We observed no effect of the task order on the kind of thoughts (TO: χ^2^ = 0.5746, p = 0.448; ZO: χ^2^ = 2.357, p = 0.154) except for a tendency for more OT thoughts when the VIMT was performed before the LDT than in the reverse order (OT: χ^2^ = 3.109, p = 0.078).

Concerning the task-order interaction, there was a tendency concerning ZO answers, more frequent during VIMT when the VIMT was passed first (χ^2^ = 3.7093, p = 0.054) and no other interaction between task and order (OT: χ^2^ = 0.5396, p = 0.463; TO: χ^2^ = 0.1439, p = 0.704).

The average proportion of responses corresponding to being off-task (“I was not thinking of anything” and “I thought of something other than the task”) is similar in both tasks: 61.3% for LDT and 61.8% for VIMT, which is coherent with the proportion of MW found in other studies (21, 25, 26).

We expected MW would be more directed toward the past (IAM) during the VIMT than during the LDT. Twenty-one subjects were not included in this analysis because they had neither thoughts toward past reported in VIMT nor in LDT. Subjects evaluated an average of 1.01 (sd = 1.21) of their thoughts as directed to the past.

There was no task effect on the proportion of past oriented tune-out thoughts among spontaneous non-perceptually driven TO thoughts (p = 0.3), (detailed results provided in **Supplementary Table 1**).

The lack of significant difference in the kind of thoughts and MW’s time orientation between the two tasks led us to gather the data collected by the thought probes of the two tasks and to consider them as a sample of the thoughts and MW of each subject for the remaining statistical analyses.

### Comparison of Dysphoric to Non-Dysphoric Subjects

The comparison was led on a total of 53 subjects for which the BDI score remained stable at the time of the experiment. The sex and level of education of the two groups were not statistically different. The dysphoric group was on average 3.7 years younger than the non-dysphoric group, the age difference was statistically significant (t = 3.4, df = 50.4, p-value = 0.001) (**Table 2**).

**Table 2:** Participant demographic and clinical characteristics.

Variable	mean D (N = 23)	mean ND (N = 37)	p	*	SD - D	SD - ND
Age	22.3	26	0.001	**	3.11	5.80
Education level	14.8	15.6	0.113	ns	1.95	1.59
BDI	22.2	2.13	<0.001	***	5.04	1.72
RRS	56	33.6	<0.001	***	9.64	8.43
RRS-Brooding	13.4	8.1	<0.001	***	3.54	2.45
RRS-Reflecting	12.5	8	<0.001	***	3.84	2.49
RRS-Depression	30.1	17.5	<0.001	***	5.83	4.95
DDFS	49.3	40.9	0.002	**	6.65	9.75

D, dysphoric; ND, non-dysphoric (t-test). *p < 0.05; **p < 0.01; ***p < 0.001. ns = not significant.

Differences between D and ND on rumination (RRS) and daydreaming (DDFS) scores groups (**Table 2**) confirmed that the populations of the two groups were different. As expected the dysphoric subjects significantly daydream and ruminate more than the non-dysphoric subjects.

There is a strong positive correlation among all participants between BDI and RRS score (R = 0.720, p < 0.001), and a weak correlation of DDFS with BDI (R = 0.354, p = 0.001) and RRS (R = 0.305, p = 0.005).

We evaluated the attentional fluctuations of the LDT and VIMT tasks from the number of errors and the variability of the reaction times. Concerning the number of errors we considered the errors by omission when the subject forgot to click or clicked after the display of a stimulus target. These errors did not differ significantly between dysphoric group and non-dysphoric group (dysphoric group: mean = 2.75, SD = 3.19, non-dysphoric group: mean = 2.05, SD = 2.8, t (51) = 0.79, p = 0.43). RT were recorded at each space bar press for the targets (N = 72 RT, one RT for each of the 36 targets × 2 tasks), RT mean was of 791 ms in the dysphoric group and 769 ms in the non-dysphoric group [t (52) = 0.54, p=0.59] (**Table 3**). In the dysphoric group, SD = 79 ms versus in the non-dysphoric group SD = 49 ms, were different (Levene test F (1,142) =15.4, p < 0.01) suggesting a higher variability of the reaction time in the dysphoric group.

**Table 3:** Group comparison, both tasks (t-tests).

	mean D (N = 23)	mean ND (N = 37)	p	*	SD - D	SD - ND
Errors	2.75	2.05	0.43	ns	3.19	2.6
RT (ms)	791	769	0.59	ns	79	49
On task	6.3	6.6	0.9	ns	4.07	4.39
Off task (TO + ZO)	11.7	11.4				
Off-task/Tune out	9.7	7.4	0.036	*	4.14	4.64
Off-task/Zone out	2.04	4.05	0.02	*	1.89	4.67

*p < 0.05. ns = not significant.

**Figure 1:**
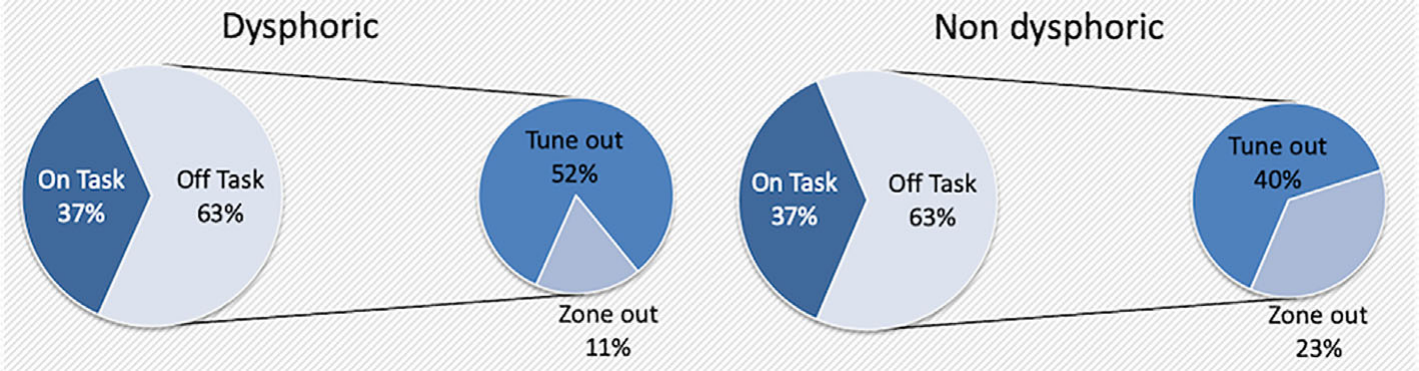
Dysphoric vs. non-dysphoric: Proportions of OT/TO/ZO.

We observed that the proportion of on-task thoughts (OT) and off-task thoughts (TO + ZO) reported by the two groups (dysphoric and non-dysphoric) is not statistically different (t = −0.07, p = 0.9) (**Table 3**). In contrast, the way of being off-task was different for the two groups. Indeed, the proportion of thoughts directed to “something else than the task (TO)” is significantly higher in the dysphoric than in the non-dysphoric group (TO_dysphoric_mean_ = 9.7, TO_nondysphoric_mean_ = 7.4, t = 2.152, df = 52.1, p = 0.036) (**Figure 1**). This result is coherent with the significant difference in the auto-evaluation by questionnaire (DDFS and RRS), where dysphoric subjects reported more daydreaming and rumination than non-dysphoric subjects (**Table 2**). Among all participants, we found a positive statistically significant Spearman’s correlation (rho) of the standardized measure of MW (DDFS) with the occurrence of TO thoughts (rho = 0.39, p < 0.0001), but not with the global occurrence of off-task thoughts (TO + ZO) (rho = 0.024, p = 0.830). No correlation was found between the standardized measure of rumination (RRS) and the occurrence of TO (rho = 0.04, p = 0.719) or global off-task thoughts (rho = −0.118, p = 0.286).

The proportions of on-task thoughts and off-task thoughts (rho = −0.096, df = 81, p = 0.389) as much as the proportion of TO thoughts (rho = 0.075, df = 81, p = 0.498) were not correlated with age.

Within the 18 thought-probes, participants reported on average 5.7 spontaneous and non-driven by perception TO thoughts. Among those, 3.4 were described as being mental time travel (MTT, IAM, and future projections), including 2 IAM. The time orientation of spontaneous thoughts did not statistically differ between the dysphoric and non-dysphoric groups, neither for IAM (past thoughts, p = 0.543) nor for global mental time travel (past and future thoughts, p = 0.752).

Among all participants, we noticed a strong correlation between the number of TO thoughts and errors committed during the tasks (rho = 0.84, p < 2.2e-16). Within TO thoughts, there was an even stronger correlation between MTT thoughts and the number of errors (rho = 0.92, p < 2.2e-16). There was no significant difference between dysphoric and non-dysphoric group.

Finally, we compared the characteristics of spontaneous thoughts, i.e., their specificity, the field or observer perspective adopted by the subject in thought and the emotional valence of the thought content between the two groups in a mixed effects model. These characteristics were not statistically different between the dysphoric and non-dysphoric subjects (specificity: t = −1.631 p = 0.109, perspective: t = 1.596, p = 0.116, emotional valence: t = −1.530, p = 0.132).

## Discussion

Our aim was to study both IAM and MW in a same group of participants with dysphoria compared to a control group, using two different tasks, with and without verbal external stimuli.

We first hypothesized that the VIMT would induce more retrieval of IAM compared to the LDT. We did not find any difference in the level of past oriented thoughts between the two tasks. However, consistent with the previous literature, we found in both tasks a high level of off-tasks responses, suggesting that both tasks were efficient to induce mind wandering in our dysphoric and non-dysphoric subjects. In order to compare the levels and feature of MW in dysphoric and not dysphoric, we thus combined the results on the two tasks.

There was a statistically significant difference of age between our two groups. Although MW has been shown to be less present in older than younger adults (27), the age difference between our groups was quite small (3.7 years) in comparison to the study of Maillet et al.(39 years), and none of the kinds of thoughts reported by participants during tasks were correlated with their age.

Contrary to our second and third hypotheses, the quantity of MW was similar in the two groups and we did not observe any significant differences in thoughts’ content characteristics (time orientation, specificity, field perspective, and emotional valence).

In line with results from other studies (19, 28), involuntary and spontaneous thoughts seems to keep their specificity in dysphoric subjects. A dissociation has been shown between voluntary and IAM retrieval in patients with MDE, with voluntary retrieval being less specific (i.e., with less spatial, temporal, and emotional details) in depressed patients with residual symptoms compared to controls (6, 7, 29, 30), and no differences between depressed patients and controls in the involuntary memories specificity on diary study (7). Plimpton et al. (20) also explored involuntary autobiographical memories with a laboratory task and found no difference in the specificity of these memories between dysphoric and non-dysphoric group. Our results are consistent with these findings suggesting that involuntary retrieval of autobiographical memories may facilitate the production of specific emotional, spatial, and temporal details. However, we should keep in mind that our participants present depressive symptoms and not the complete symptomatic criteria for major depression, thereby may not have the same repercussion on IAM and voluntary autobiographical memory retrieval. A study in both acute depressed patients and dysphoric subjects are clearly needed with tasks comparing directly the voluntary and involuntary retrieval of autobiographical memories.

The focus during MW was different between groups. When they presented dysphoria, participants appeared more focused on their task-unrelated thoughts than controls and they had more access to its contents (more TO and less ZO than controls). This difference may suggest a greater attentional shift during MW for dysphoric subjects than controls. Consistent with this interpretation, the greater variability of response time during both tasks in participants with dysphoria points out more variability in sustained attention in this population compared to controls.

Smallwood and Andrews-Hanna (31) make two assumptions about the occurrence and regulation of MW. First, according to the *content regulation hypothesis*, the subject is able to regulate the content of self-generated thoughts in order to maximize the current perceptual experience. According to this approach, in dysphoria, the dysregulation of MW is due to an attention of the subject captured by its (essentially negative) personal concerns. MW episodes, particularly when turned toward the past, lead to an increased negative mood which can activate processes of rumination (emotional pathway). Secondly, the *context regulation hypothesis* assumes that the subject is able to regulate the occurrence of his self-generated thoughts in order to optimize his cognitive performance when performing a task. According to this hypothesis, in dysphoria, the intensity of MW, through the attentional decoupling that it implies, would negatively affect the cognitive capacities and the performance of the subject (cognitive pathway).

Our results concerning the increased variability of response time and the greater attentional shift during MW (greater focus on MW), in dysphoric group, in line with other studies (25, 32) support the hypothesis of a MW dysregulation starting from a cognitive pathway. These results support the *context regulation hypothesis* in dysphoric subjects. The absence of significant difference of MW content characteristics (time orientation, emotional valence, and perspective), is also in favor of this hypothesis.

Recent works (33, 34) highlight the concept of *meta-awareness* (being aware of our current thinking) in the study of MW. Drescher et al. (34) explored MW, meta-awareness, and cognitive control by three distinct tasks and showed that subjects with a higher meta-awareness had a better cognitive control but detected less their MW. We must be cautious interpreting our results concerning the 1^st^ answer (on task/tune out/zone out) to the thought probes, regarding this concept of meta-awareness. Concerning the “zone-out” events: the mention “I was not thinking about anything” is not precisely “I don’t know what I was thinking”. Subject may have interpreted it as “I’m doing the task” as opposed to the first proposal “I’m thinking about the task”. We cannot be certain whether if “zone-out” events were MW without meta-awareness rather than an absence of MW. We also don’t have information about “tune-out” events being MW with or without meta-awareness before the thought-probe asking. Further studies are clearly needed to explore meta-awareness in dysphoria and depression.

Meta-awareness and the cognitive “context” dysregulation of MW are important to consider in the management of subjects at risk for depression. Several studies already showed the effectiveness of Mindfulness Based Cognitive Therapy for preventing depressive relapse (35). This therapy targets attention to the present moment, and meta-awareness of one’s thoughts flow, independently of the content of MW.

Other studies are exploring the use of transcranial current direct stimulation on specific areas like LPFC (36) to modulate MW. Overall, both therapeutic tools could be explored for prevention of a depressive episode in a population presenting dysphoria.

## Conclusion

In summary, we assessed simultaneously IAM and MW in participants with dysphoria and controls. Participants with dysphoria appeared more focused on their task-unrelated thoughts than controls; they had more access to its contents. Moreover, they showed a greater variability in time reaction. These results suggest a difficulty to regulate the occurrence of self-generated thoughts rather than their content that may confer to dysphoric subjects decreased cognitive ability. This dysregulation may partly underlie the cognitive symptoms of depressive disorder and therefore could constitute a target for further preventive intervention.

## Data Availability Statement

The raw data supporting the conclusions of this article will be made available by the authors, without undue reservation.

## Ethics Statement

The studies involving human participants were reviewed and approved by the ethics committee of Ile-de-France VI on March 13, 2013, CNRS protocol no. 12014. The patients/participants provided their written informed consent to participate in this study.

## Author Contributions

AG was involved in the conception of the work, the data collection, the data analysis and interpretation, and drafting the article. F-XL contributed to the data analysis plan and application and revising the statistics part of the article. J-YR made a critical revision of the article. NG was involved in the analysis plan and the critical revision of the article. PF supervised the conception of the work, directed the project, and made several revisions of the article. AG, F-XL, J-YR, NG, and PF approved the final version of the article.

## Funding

This work was financed by recurrent funding from the ICM.

## Conflict of Interest

The authors declare that the research was conducted in the absence of any commercial or financial relationships that could be construed as a potential conflict of interest.
