# Supplementary figures and images for: Mind-Wandering Changes in Dysphoria
Source: Front Psychiatry. 2020 Sep 11;11:544999. doi: 10.3389/fpsyt.2020.544999 (PMC7533624; doi:10.3389/fpsyt.2020.544999)

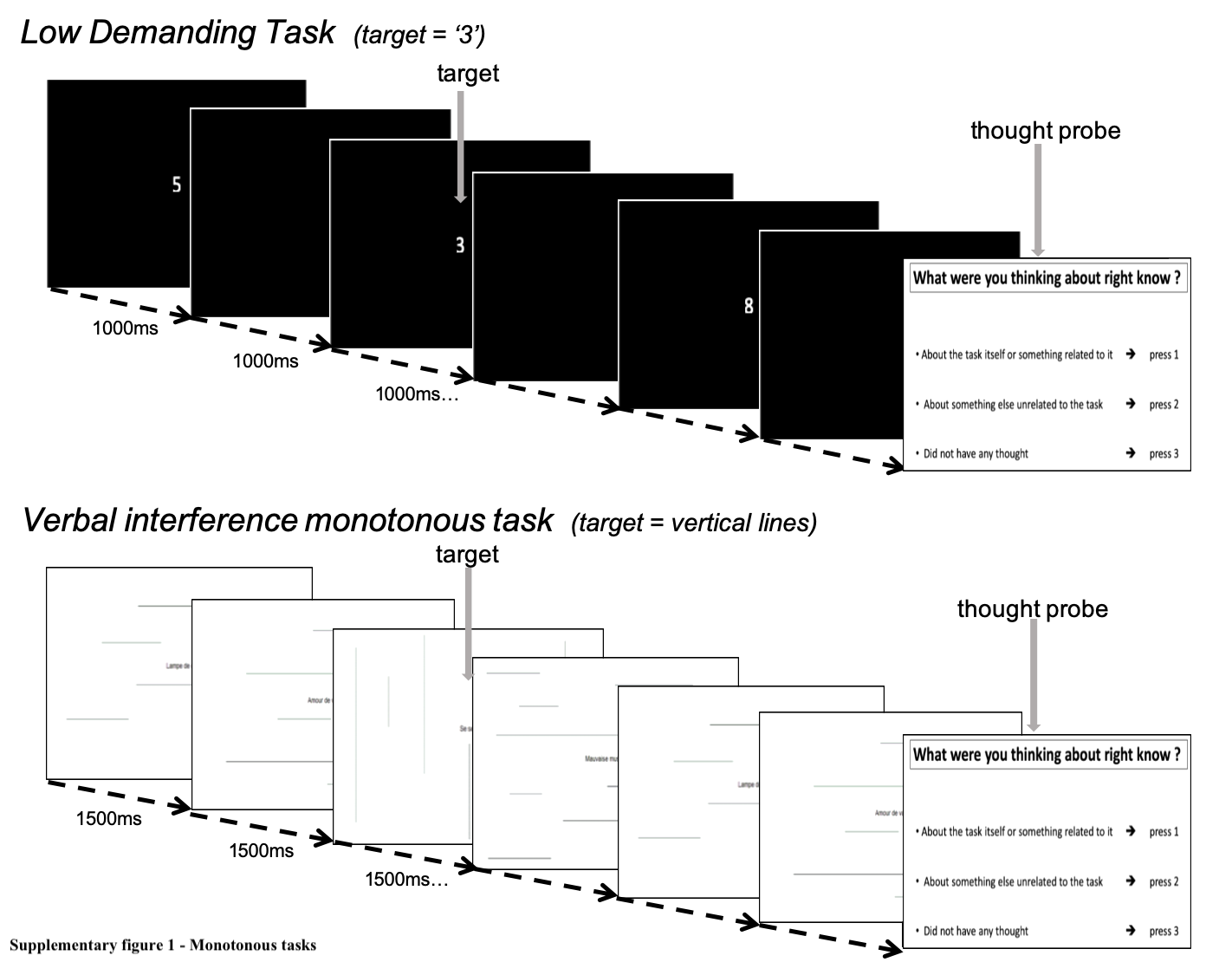

Supplement: Supplementary file 1 [file Image_1.png]

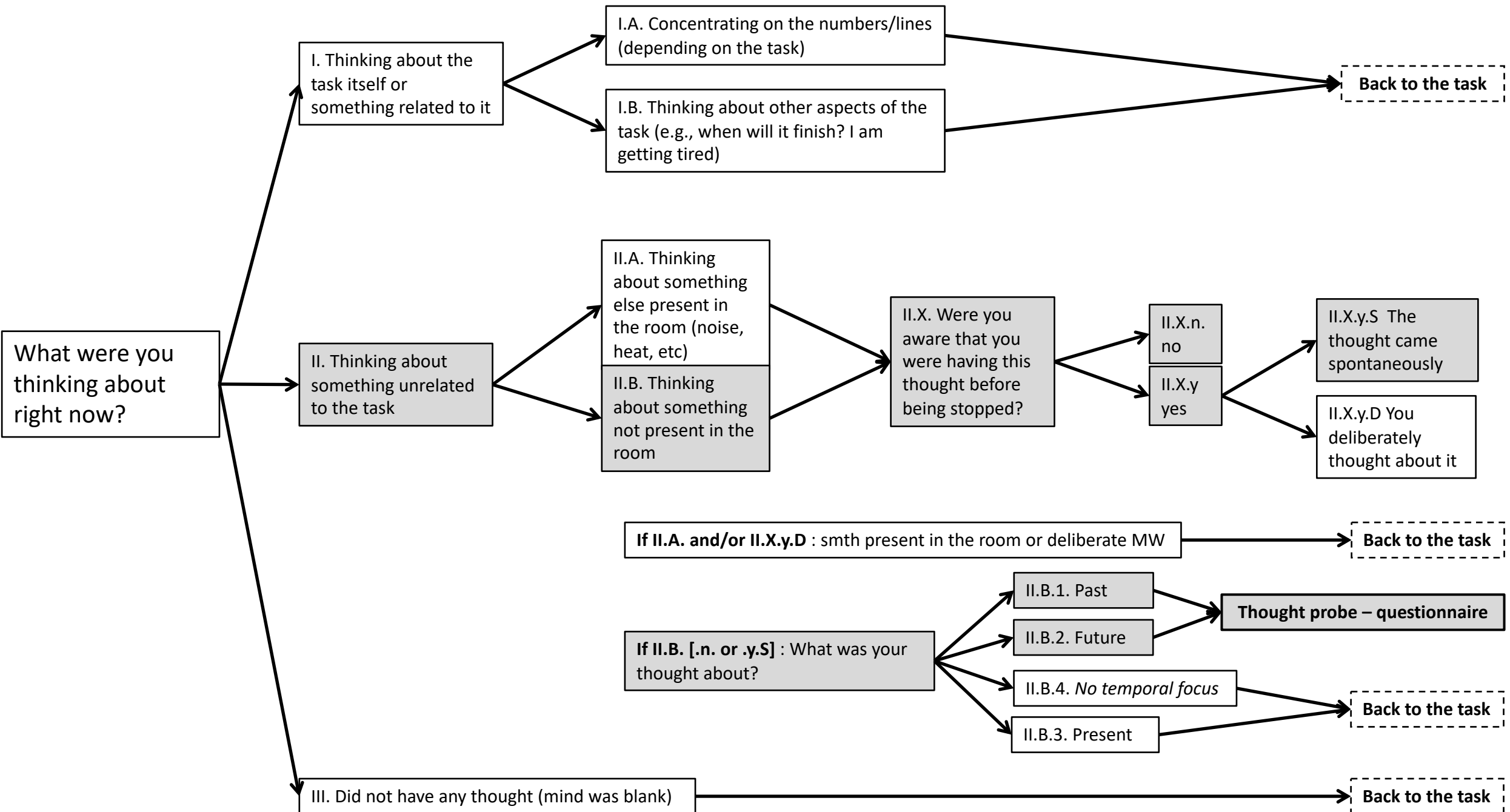

### Supplementary figure 2. Thought probe - structure

Supplement: Supplementary file 2 [file Image_2.pdf]
