# Supplementary material for: Mind-Wandering Changes in Dysphoria
Source: Front Psychiatry. 2020 Sep 11;11:544999. doi: 10.3389/fpsyt.2020.544999 (PMC7533624; doi:10.3389/fpsyt.2020.544999)
Supplement: Supplementary file 3 [file Image_3.pdf]

Questionnaire if thought not task related, spontaneous and directed towards past or future :

1. At **which moment** of the past *(or future)* does the content of the thought take place?
   a. Less than one year ago (In less than one year)
   b. Between 1 and 5 years ago (In between 1 and 5 years)
   c. More than 5 years ago (In more than 5 years)

2. **Specificity :**
   a. Did it happen only once? *(Would it…)* Yes/no
   b. Did it last less than 1 day? *(Would it…)* Yes/no
   c. Was it located in time and space? *(Would it be…)* Yes/no
   d. Can you remember at least 2 specific details about it? *(Do you picture…)* Yes/no
   e. How well can you picture the thought ?

   Very vaguely → Very accurately, like if it was normal vision

3. In your thought, what **visual perspective** did you have?

   Third person perspective → Blended perspective → First person perspective

4. How **pleasant** was your thought?

   Very unpleasant → Very pleasant

Supplementary figure 3. Thought questionnaire : questions asked to the participants when a thought is unrelated to the task, spontaneous and either future or past oriented
