# Supplementary material for: Mind-Wandering Changes in Dysphoria
Source: Front Psychiatry. 2020 Sep 11;11:544999. doi: 10.3389/fpsyt.2020.544999 (PMC7533624; doi:10.3389/fpsyt.2020.544999)
Supplement: Supplementary file 4 [file Table_1.pdf]

Supplementary table 1

Involuntary Autobigraphical Memory : **Percentage of past-oriented thoughts: past/(past+present+future+NT)*100**

NA's: subjects with no reported past thoughts

		Both tests combined						
**Group**	**N**	**Min.**	**1st Qu.**	**Median**	**Mean**	**SD**	**3rd Qu.**	**Max.**
ND	35	0	9,2	33,3	33,8	28,0	50	100
D	21	0	14,3	25	27,6	40	63	0

ND vs D: Wilcoxon-Mann-Whitney: W = 385, p-value = 0.543

		SART								VIMT							
**Group**	**N**	**Min.**	**1st Qu.**	**Median**	**Mean**	**SD**	**3rd Qu.**	**Max.**	**NA's**	**Min.**	**1st Qu.**	**Median**	**Mean**	**SD**	**3rd Qu.**	**Max.**	**NA's**
ND	35	0	0	33,3	31,2	29,9	50	100	4	0	0	33,3	33,9	34,6	50	100	3
D	21	0	20	21,8	33,3	100	0	0	10,7	22,5	33,1	51,4	100	1	52,8	100	1

ND vs D: Wilcoxon-Mann-Whitney: W = 373.5, p-value = 0.209 Wilcoxon-Mann-Whitney: W = 289, p-value = 0.773

Mental time-travel **Percentage of either past- or future-oriented thoughts: (past+future)/(past+present+future+NT)*100**

NA's: subjects with no reported travel time thoughts

		Both tests combined						
**Group**	**N**	**Min.**	**1st Qu.**	**Median**	**Mean**	**SD**	**3rd Qu.**	**Max.**
ND	35	0	40,0	57,1	56,1	30,9	75	100
D	21	0,0	45,5	55,6	8923933	71,4	100,0	0,0

ND vs D: Wilcoxon-Mann-Whitney: W = 368.5, p-value = 0.752

		SART								VIMT							
**Group**	**N**	**Min.**	**1st Qu.**	**Median**	**Mean**	**SD**	**3rd Qu.**	**Max.**	**NA's**	**Min.**	**1st Qu.**	**Median**	**Mean**	**SD**	**3rd Qu.**	**Max.**	**NA's**
ND	35	0	16,7	66,7	56,1	39,6	100	100	4	0	33,3	55	59,3	34,6	100	100	3
D	21	20,0	50,0	48,5	71,4	100,0	0,0	0,0	38,3	60,0	56,5	77,1	59,5	30,2	79,2	100	1

ND vs D: Wilcoxon-Mann-Whitney: W = 356, p-value = 0.372 Wilcoxon-Mann-Whitney: W = 298.5, p-value = 0.922

ND = Non Dysphoric

D = Dysphoric
